# Towards a new taxonomy of preterm birth

**DOI:** 10.1038/s41372-024-02183-z

**Published:** 2024-11-20

**Authors:** David K. Stevenson, Alan L. Chang, Ronald J. Wong, Jonathan D. Reiss, Brice Gaudillière, Karl G. Sylvester, Xuefeng B. Ling, Martin S. Angst, Gary M. Shaw, Michael Katz, Nima Aghaeepour, Ivana Marić

**Affiliations:** 1 Department of Pediatrics, Division of Neonatal & Developmental Medicine, Stanford University School of Medicine, Stanford, CA USA; 2 Department of Anesthesiology, Perioperative and Pain Medicine, Stanford University, School of Medicine, Stanford, CA USA; 3 Department of Biomedical Data Science, Stanford University, Stanford, CA USA; 4 Department of Surgery, Stanford University School of Medicine, Stanford, CA USA

**Keywords:** Predictive markers, Paediatrics

## Abstract

Disease categories traditionally reflect a historical clustering of clinical phenotypes based on biologic and nonbiologic features. Multiomics approaches have striven to identify signatures to develop individualized categorizations through tests and/or therapies for ‘personalized’ medicine. Precision health classifies clinical syndromes into endotype clusters based on novel technological advancements, which can reveal insights into the etiologies of phenotypical syndromes. A new taxonomy of preterm birth should be considered in this context, as not all preterm infants of similar gestational ages are the same because most have different biologic vulnerabilities and hence different health trajectories. Even the choice of interventions may affect observed clinical conditions. Thus, a new taxonomy of prematurity would help to advance the field of neonatology, but also obstetrics and perinatology by adopting anticipatory and more targeted approaches to the care of preterm infants with the intent of preventing and treating some of the most common newborn pathologic conditions.

Clinicians and scientists appreciate that categories of different diseases as described in the medical literature reflect a historical clustering of clinical phenotypes that seem to resemble each other based on observable features. For example, patients with an excessively high blood glucose level have been diagnosed as having diabetes mellitus even though there are many other conditions that result in hyperglycemia [1] and require various therapeutic approaches designed to resolve it [2–5]. Diabetes mellitus is not a single disease simply explained by one particular gene mutation or a perturbation in a single biochemical pathway [1]. Indeed, every human condition, even the “single gene” defects, can present as syndromes with considerable clinical phenotypic variations. This does not mean that generalizations of diagnostic and therapeutic approaches to individuals with similar phenotypes are inappropriate, but it also should not amaze us that individuals do respond differently to the treatments that we administer. The use of polygenic risk scores (PRS) and other effective and sophisticated computational techniques has helped us understand and identify the diversity of endotypes. Endotypes are specific subclassifications of diseases or syndromes and are defined by particular pathophysiologic mechanisms and associated clinical biomarkers. Their existence has not been appreciated fully by traditional diagnostic methods, but was suggested by tremendous variations in the responses to our usual biologic interventions, which often assumed a shared causation [6].

While PRS have shown promise in predicting risk for certain adult conditions, their applicability in neonates is limited. Primary challenges include the significant biological variability in neonates and the need for large sample sizes to achieve reliable predictive power. As such, we propose establishing global collaborations that pool data across healthcare systems and regions (both high- and low-resource settings), which could help amass a sufficiently large and diverse dataset. This would enhance the validity of PRS in neonates and potentially uncover novel, population-specific risk factors. This approach could form the foundation for globally representative neonatal PRS, making risk prediction more accurate and accessible worldwide.

## Genomics approaches

Genomics approaches strive to understand signatures to develop individualized tests and therapies fulfilling the expectation of personalized medicine. The extensive complexity of the human genome is unique for each person and makes this a promising goal but a daunting task. Advances in machine learning (ML) and artificial intelligence (AI) provide a set of tools that are increasingly being used to achieve this vision. Another complexity, even for diseases with a strong hereditary component, is a patient’s lifestyle that can modify risk of disease and requires consideration of genomics (as well as other omics) and exogenous features for better prediction. Several successes for complex disease risk prediction have been reported. By using sparse ML modeling and integration of whole genome and electronic health records (EHR), an accurate model for risk prediction of abdominal aortic aneurysm (AAA) development has been created [7]. The sparse model identified 60 genes that are most predictive of AAA development and further enabled identification of functional modules and pathways. These results could potentially lead to development of a clinical test for AAA. Deep-learning methods have also been applied to the analysis of genomics data: the use of auto-encoders for prediction modeling has been shown to improve accuracy in cancer detection in contrast to more traditional ML models (e.g., principal component analysis) [8–11], because of their ability to improve capture of non-linear relationships. New data sources linking biobanked genetic with EHR data may further facilitate integration of genetic and EHR information for better prediction of polygenic risk [12].

To enhance the generalizability of genomics findings, incorporation of a broad spectrum of racial, ethnic, and geographic backgrounds is crucial, as outcomes can vary significantly across these groups. Currently, many genomics (and other omics) studies and prediction models from clinical/EHR data are based on populations of European descent, which can limit their relevance for other groups. Therefore, research initiatives must be prioritized to actively recruit diverse populations. Additionally, while training ML algorithms on diverse datasets is essential, it alone is insufficient. These datasets should reflect an inclusive demographic range and data collection methods should be culturally and contextually sensitive to minimize biases.

The application of genomics and large datasets to delineate neonatal risk factors offers substantial benefits beyond risk prediction, particularly in the context of clinical trials. By identifying specific high-risk subpopulations, genomics data can enrich study cohorts, focusing resources on those most likely to exhibit measurable responses to interventions. This can significantly reduce sample sizes needed to achieve statistical power, lower trial costs, and shorten study duration. Such benefits are critical in neonatal research, where recruitment and targeted study conditions may be rare.

## Beyond genomics approaches and towards a new taxonomy of preterm birth

The integration of multiomics, (such as genomics, epigenomics, transcriptomics, proteomics, lipidomics, metabolomics, immunomics, and microbiomics) datasets combined with novel ML and AI methods are revolutionizing our understanding of complex human conditions and diseases [13, 14]. Also, discoveries in one specific area, such as in cancer research, may have relevance in others, such as autoimmunity [15], cardiology [16], and aging [17, 18]. Indeed, the same reasoning can be extended to the various syndromes associated with pregnant women and neonates. As one prominent example, pregnancy and the onset of labor are phenomena with immunological involvement, as indicated by the role of regulatory T cells in immune tolerance throughout pregnancy [19], peripheral immune profile shifts throughout pregnancy progression [20], the implications of pregnancy-induced microchimerism of the woman’s immune system [21], and work that demonstrates the ability to time the onset of labor using immunological profiling [22]. One additional implication of the immunological involvement in pregnancy is that the extent of immune reaction to the fetus, as related to the classical idea of fetal tolerance, may have significant impact on the inflammatory status of the newborn at delivery and subsequent risk of adverse outcomes. Indeed, bronchopulmonary dysplasia (BPD), the most common acquired disorder of extreme prematurity, is driven in part by a localized inflammatory milieu within the still-developing lung. In addition, necrotizing enterocolitis (NEC) evolves secondary to perturbations in inflammatory cascades within gut enterocytes. Finally, neonatal sepsis, both early- and late-onset, is fundamentally driven by immaturities and imbalances in innate and adaptive immune components.

A significant challenge to longitudinal neonatal outcome studies in the US is the frequent movement of individuals across healthcare systems, which complicates data linkage between maternal, neonatal, and childhood phases. This underscores the critical need for greater interoperability of EHRs and standardization of diagnostic terminologies. For instance, BPD currently lacks a uniform definition with many different criteria in use. Such variation hinders reliable assessments of long-term outcomes. Thus, a cohesive taxonomy that integrates standardized definitions and interoperable data structures is essential to track and interpret complex health trajectories effectively.

## The case of preterm birth

Preterm birth is a syndrome with many etiologies that are influenced by biological and socioeconomic factors [23, 24], although there may be common pathways for parturition, which can be identified and predicted with the discovery of strong biomarkers. Thus, there are various endotypes of preterm birth that underlie its heterogeneity, which are likely reflected in the phenotypes of preterm infants and the conditions they develop after birth. Certainly, there are developmental features that contribute to the disabilities that we observe in neonates at different gestational ages and weights, but not all neonates of the same gestational age or weight are alike biologically either. They may have different biologic dispositions after birth and can be inferred to have different biologic predispositions before birth related to their mother’s biologic disposition or the combination of the mother’s, fetus’s, and placenta’s cellular signaling interactions. Thus, information about the maternal, fetal, and placental circumstances based on various omics measures alone [25–29] or in integrated combination with EHRs can predict health trajectories of not only the mother, but even of the infant after birth [30, 31].

The increase in preterm birth rates among immigrant women from countries with lower rates highlights the potential role of environmental factors, including epigenetic influences, in impacting outcomes. Factors such as obesity, which has been associated with higher risks of preeclampsia and potentially preterm delivery, likely interact with genetic predispositions. To incorporate these influences, our approach can be enhanced by integrating data on environmental and lifestyle factors, such as changes in body mass index, diet, and psychosocial stressors, along with epigenetic profiles where available. This multi-layered approach allows us to capture how exposure to new environments might activate or suppress genetic pathways associated with preterm birth, providing a more comprehensive risk assessment.

## Future directions

Therefore, in the practice of scientific medicine, the focus is changing from where a biologic system has been to where it is headed – from a concentration-based approach to diagnosis followed by a reactionary-based therapeutic response aimed at returning the measure back to a ‘normal’ level. For example, patterns of omics measures or simple ratios of selected factors, could indicate that a biologic system may be headed in a pathophysiologic direction or measuring changes in various biologic features over time could suggest the same thing. Thus, predicting health trajectories for individuals, pregnant women, fetuses, neonates, children, or adults has become possible. The applications of ML and AI have contributed to the feasibility of using such approaches. It is crucial to expand these efforts beyond the most highly-resourced settings: in recent collaborative work, the combination of broad omics assay technologies and AI has been shown to empower a deep understanding of the biology of preterm birth across several sites in low- and middle-income countries (LMICs) [32, 33]. For LMICs where the prevalence of undesirable pregnancy outcomes, such as preterm birth and preeclampsia is the highest [24], development of point-of-care, low-cost diagnostic tools for prediction of these risks, which are affordable in these settings, could drastically reduce morbidity and mortality. Availability of many omics measurements, characterizing in detail biological changes in pregnancy, can provide data for the development of such tests. Systematic analysis of these high-dimensional data using sparsity-promoting ML techniques [34] has proved capable of identifying a small number of highly predictive biomarkers that could serve as a basis for such tests [28, 35]. A common challenge in a clinical setting comes from having to train an ML algorithm on a relatively small sample number. Consequently, small perturbations in training data yield different sets of selected biomarkers, questioning their robustness. A recently developed sparsity-promoting regularization method, Stabl, overcomes this obstacle enabling better translation of such ML results to practice [36]. Also, it is important to remember that ML algorithms should be trained in ways to avoid unintended biases that could confound their findings in different geographical or cultural settings.

In general, we are learning to appreciate the robustness of living systems with redundancies that have evolved over time to adapt to the stresses encountered. However, we must also consider that acquired complications of prematurity have not been fully subject to evolutionary pressures owing in part to recent successes in neonatology for survival of those born prematurely. In addition, we must also consider how different pathological states and the variety of phenotypes that we observe in medicine (within a traditionally defined disease category) depend not only on our genetic capacities, but also on what genes are expressed, when they are, and by how much. Taken together, life is a symphony of chemical reactions, and the pathologies of life are like cacophonous compositions played by an “incompetent” orchestra or led by a ‘tone-deaf’ conductor. Moreover, we are learning that health trajectories over a lifetime may be influenced or even established early on by maternal-fetal interactions and capable of impacting newborn dispositions, as cellular responses are programmed early due to exogenous circumstances. For the future, the use of wearable devices to provide continuous health monitoring can also be used for such predictions, indicating the need to intervene and potentially correct the trajectory of a biologic system in a timely fashion, even before clinical signs and symptoms become apparent. One example of this is in recent work where algorithmic analysis of sleep and activity tracking of women during pregnancy revealed subgroups of individuals that were at heightened risk of preterm birth [37]. Wearable technologies are also promising points of patient intervention to leverage in the settings of post-partum depression [38] and labor onset to determine admission time [39].

Finally, although long-term projections of health and early interventions to promote robust health spans are laudable goals, much shorter timeframes for medical decision-making also require anticipatory guidance to avoid catastrophes, like multisystem organ failure in shock and irreversible injury to the brain, intestines, or heart – or even death. Such are the circumstances in emergency rooms, operating rooms, and neonatal intensive care units. In these environments, live-streaming data monitors, providing real-time information about a variety of bodily functions, may provide the data sources needed for timely short-term decisions about various clinical maneuvers, such as changes in ventilator settings, oxygen administration, administration of volume, and the use of cardiotonic medications [40, 41]. Again, computer algorithms may be able to identify patterns that can quickly inform and guide physicians toward making the right choices and stabilize a patient’s physiology and avoid clinical deterioration. Algorithmic approaches have already made major strides in the information processing of bedside monitoring data for integrated representation learning of sleep across multiple modalities [42] and cardiologist-level analysis of ambient electrocardiograms [43]. Furthermore, reinforcement learning can be of value in personalized treatment strategies and clinical decision support [44].

## Conclusions

The case for a new taxonomy of preterm birth should be clear to neonatologists: not all preterm infants of a similar gestational age are the same; some are more mature than others despite their shared chronology; and some have different biologic vulnerabilities. That is, they are all disposed differently because of their specific fetal circumstances and predispositions. These differences set them on different health trajectories, which can expose them to medical interventions, which, in themselves, can reveal further differences in their biological responses. Indeed, sometimes through their choice of interventions, physicians may even contribute often unwittingly or unavoidably to observed clinical conditions, such as BPD, NEC, intraventricular hemorrhage, and retinopathy of prematurity [45–48]. Nonetheless, having a better sense of the endotypes of preterm infants, through a biology-based taxonomy of prematurity that considers more than just gestational age, would help to advance the field of premature newborn care by allowing physicians to adopt an anticipatory and more targeted approaches to the care of preterm infants with the possibility of preventing some of the most common newborn conditions. A rewriting of some of the current definitions of disease may be necessary to fully capture the heterogeneity of the developmentally premature newborn through the power of omics technologies. This holds the potential to reduce outcome misclassification, better identify risk factors, increase diagnostic and therapeutic yields, and boost power in clinical trials. The future of precision health for neonates is closer than it ever has been before, with some of the benefits being better predictive, more preventive, and less expensive neonatal healthcare. A further benefit might be healthier adults with longer, productive lives.

**Fig. 1:**
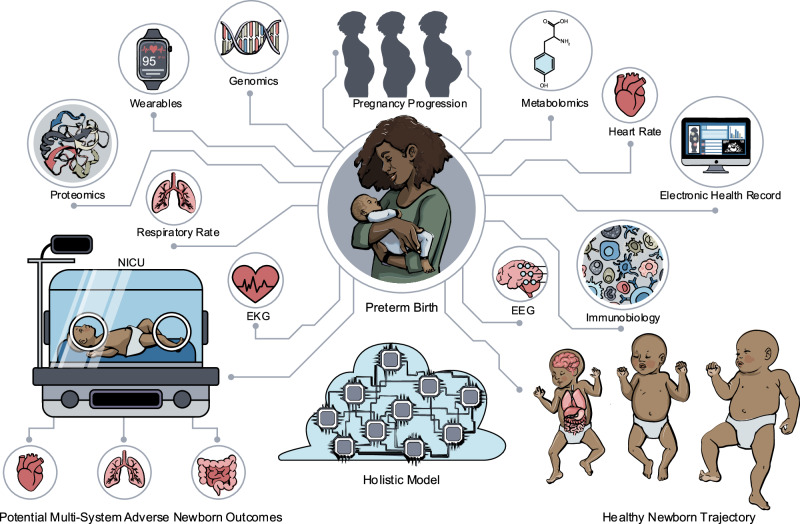
A new data-driven taxonomy of prematurity expressed throughout pregnancy and early life. Preterm infants of similar gestational ages and birthweight are not all the same – some are more mature than others despite shared chronology and all have different biological vulnerabilities that can affect multiple organ systems. Thus, a more complete taxonomy of prematurity is needed that could track infant prognosis throughout pregnancy and in the peripartum period through a data-driven lens with full consideration of the maternal-fetal dyad throughout gestation and after delivery. The current scale of biological assay technologies to interrogate the immune system, genome, proteome, and metabolome has coincided with more robust data infrastructure for EHRs in order to enable integrative AI approaches in the obstetric and neonatal domains. Furthermore, wearable technologies have also entered into the domain as a way to understand the health status on a second-by-second basis. This data-driven approach is also readily extendable to the neonatal intensive care setting, as the real-time read-outs provided by data monitoring systems may provide additional insights needed for timely healthcare decisions regarding changes in ventilator settings, oxygen administration, the use of cardiotonic medications, and other time-sensitive clinical maneuvers.
